# Optimal Cloning of PCR Fragments by Homologous Recombination in *Escherichia coli*

**DOI:** 10.1371/journal.pone.0119221

**Published:** 2015-03-16

**Authors:** Ana Paula Jacobus, Jeferson Gross

**Affiliations:** Department of Biological Sciences, University of São Paulo, Luiz de Queiroz College of Agriculture, Piracicaba, São Paulo, Brazil; Saint Louis University, UNITED STATES

## Abstract

PCR fragments and linear vectors containing overlapping ends are easily assembled into a propagative plasmid by homologous recombination in *Escherichia coli*. Although this gap-repair cloning approach is straightforward, its existence is virtually unknown to most molecular biologists. To popularize this method, we tested critical parameters influencing the efficiency of PCR fragments cloning into PCR-amplified vectors by homologous recombination in the widely used *E*. *coli* strain DH5α. We found that the number of positive colonies after transformation increases with the length of overlap between the PCR fragment and linear vector. For most practical purposes, a 20 bp identity already ensures high-cloning yields. With an insert to vector ratio of 2:1, higher colony forming numbers are obtained when the amount of vector is in the range of 100 to 250 ng. An undesirable cloning background of empty vectors can be minimized during vector PCR amplification by applying a reduced amount of plasmid template or by using primers in which the 5′ termini are separated by a large gap. DpnI digestion of the plasmid template after PCR is also effective to decrease the background of negative colonies. We tested these optimized cloning parameters during the assembly of five independent DNA constructs and obtained 94% positive clones out of 100 colonies probed. We further demonstrated the efficient and simultaneous cloning of two PCR fragments into a vector. These results support the idea that homologous recombination in *E*. *coli* might be one of the most effective methods for cloning one or two PCR fragments. For its simplicity and high efficiency, we believe that recombinational cloning in *E*. *coli* has a great potential to become a routine procedure in most molecular biology-oriented laboratories.

## Introduction

Recombinant DNA techniques are indispensable in any modern laboratory that relies on molecular biology methods. Traditionally, molecular cloning depends on restriction endonucleases to produce linear vectors and inserts that are fused by the DNA ligase [1]. Recombinant DNA technology has also been greatly facilitated by the use of Polymerase Chain Reaction (PCR). A standard DNA cloning practice is to amplify a DNA of interest with oligonucleotides containing 5′ tails specifying endonuclease recognition sites. These allow subsequent cleavage and insertion of PCR fragments into any desired cloning vector. Notwithstanding the universality of these traditional cloning approaches, the preparation of reactant molecules with restriction enzymes and their combination by DNA ligase involves many steps, being not only laborious, but often rendering meager or frustrating results. To overcome such limitations, several PCR-based cloning protocols have proposed to skip the use of DNA ligase and also dispense the need for restriction endonucleases [1]. Among them, homology-based methods take advantage of PCR products flanked on both sides by 15 to 60 bp long sequences that perfectly match the ends of a linear vector [1]. This is facilitated by PCR amplification of fragments with oligonucleotides containing 5′ appendages homologous to the cloning plasmid. After fragment preparation, the cloning reaction can be driven *in vitro* by enzymatically-assisted recombination between the vector and the PCR inserts (e.g., SLiCE [2] and Gateway [3] methods). Alternatively, the PCR fragments and linear plasmid can be enzymatically treated to generate single-stranded DNA (ssDNA) on their termini (e.g., SLIC [4] and USER [5] methods). The resulting vector and insert overhangs are complementary and hybridize in a double-stranded DNA (dsDNA) formation containing a nick. During transformation in *E*. *coli*, the nick is repaired *in vivo*, sealing a perfect circular recombinant plasmid. Other homology-based cloning procedures rely on a typical PCR setting in which the primers have been omitted (e.g., OEC [6] and CPEC [7] methods). Following the heat denaturation step, overlapping free termini of vector and PCR fragments can anneal by base pair complementarity. The hybrid regions then serve as “megaprimers” in the extension step catalyzed by the DNA polymerase. As a result, a nicked recombinant plasmid is generated that is repaired *in vivo* in *E*. *coli*. Besides those recent innovations, traditional gap-repair in yeast stands as one of the most effective homology-based cloning approaches [8,9]. This procedure consists of merely co-transforming yeast cells with a mixture of the linear vector and PCR fragments, both having common flanking sequences. Following transformation, the yeast DNA repair machinery is very effective to recombine vector and insert homologous termini, generating a closed plasmid.

Interestingly, an analogous *in vivo* gap-repair cloning method in *E*. *coli* was described by two groups as early as 1993 [10,11]. The principle of this cloning approach in *E*. *coli* is equally as simple as in yeast. By co-transforming a linear vector and PCR fragments containing homologous ends, several *E*. *coli* laboratorial strains are capable of recombining the reactants *in vivo*, generating a closed propagative plasmid. Although this technique is simpler than traditional ligase-dependent cloning, only a few published reports using gap-repair cloning in *E*. *coli* exist [12–17]. Some studies elaborate this cloning principle in hyper-recombinogenic *E*. *coli* strains in which the RecE and RecT homologous recombination pathway is over-expressed (the ET cloning method) [12]. A few other published reports propose specific applications for gap-repair cloning in *E*. *coli*. For example, homologous recombination was successfully used for high-throughput cloning of 1302 ORFs amplified from the *Campylobacter jejuni* genome [13]. The strategy was to supply PCR fragments with 21 bp flanks identical to the ends of a cloning vector prepared with restriction enzymes. Similarly, Klock *et al*. described the gap-repair cloning of 448 full-length and 2143 truncated ORFs from 30 different bacterial sources [14]. However, in this case, both the vector and the ORFs were amplified by PCR. The authors proposed that a different mechanism than *in vivo* homologous recombination explained the obtained results. It was suggested that in normal PCR reactions, a large fraction of amplified fragments have ssDNA stretches on both termini due to incomplete primer extension during the PCR elongation step (therefore the method was termed PIPE, Polymerase Incomplete Primer Extension) [14]. Upon mixture, vector and insert complementary ssDNA regions anneal and a single recombinant DNA molecule is sealed *in vivo* following transformation in *E*. *coli*. The efficacy of gap-repair cloning through the PIPE protocol has been recently confirmed in a study in which this procedure was compared with the SLIC and OEC techniques [15]. The FastCloning method is another recombination-mediated approach that is very similar to the PIPE protocol [16]. It describes a quick and efficient way of cloning PCR fragments containing 16 bp flanking regions overlapping the ends of a linear cloning vector. Both fragments and vectors are first obtained by PCR. A mixture of vector and insert is subsequently treated with DpnI to eliminate the template plasmids background and then readily transformed in *E*. *coli* to obtain the desired clones. In line with the PIPE description, the authors of the FastCloning protocol speculate that the cloning mechanism relies on vector and inserts having complementary ssDNA overhangs that are fortuitously generated by the DNA polymerase 3′ to 5′ exonuclease activity [16].

The examples described above illustrate that recombinational cloning in *E*. *coli* is a highly efficient technique that presents remarkable advantages over conventional cloning with restriction endonucleases and DNA ligase. However, it is puzzling that this molecular cloning principle has so far been ignored by a wider community of molecular biologists. Not only is the amount of publications reporting the use of gap-repair in *E*. *coli* very little, but the interpretation of the cloning mechanism itself has been contradictory between them [10,11,14,16]. In an attempt to unify those different reports and to popularize recombinational cloning in *E*. *coli* as a practical method, we present here a study of the different parameters that modulate the efficiency of this technique. We also provide evidence dismissing the assumption that ssDNA complementary tracts, supposedly generated on the vector and inserts by DNA polymerase incomplete primer extension [14] or exonuclease activity [16], are critically needed for gap-repair cloning to occur in *E*. *coli*. Finally, to support the notion that recombination cloning in *E*. *coli* might be the method of choice for assembling single and double PCR fragments into a plasmid, we optimized the cloning parameters to obtain 94% efficiency during the construction of five different recombinant DNAs.

## Materials and Methods

### PCR amplification of vector and inserts

**Fig 1:**
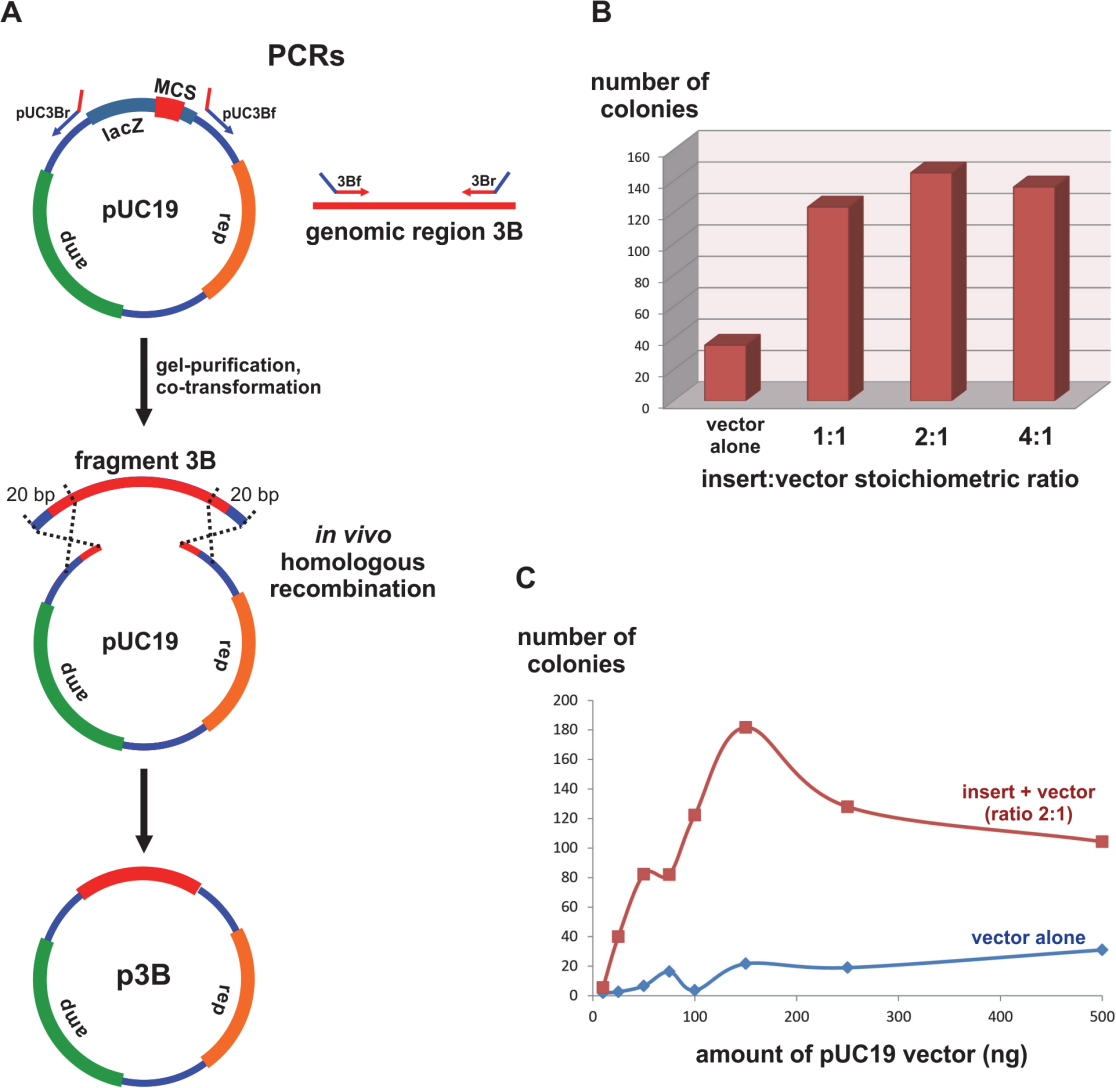
Schematic view of recombinational cloning and the influence of inserts and vector stoichiometry and quantity. (A) The plasmid pUC19 was linearized by PCR using the primers pUC3Bf and pUC3Br, which contained short 5′ overhangs (red) matching both ends of the fragment 3B. In parallel, the *S*. *japonicus* genomic region 3B was amplified by PCR using the primers 3Bf and 3Br, specifying 5′ short appendages (blue) overlapping the pUC19 vector ends. After gel-purification, vector and fragments were mixed and co-transformed in *E*. *coli*. Sequence tracts of 20 bp shared by the vector and fragment were substrates for a homologous recombination reaction resulting in the plasmid p3B. (B) *E*. *coli* competent cells were transformed with a fixed amount of 100 ng of the linear pUC19 vector mixed with a variable stoichiometric amount of the fragment 3B. The colony numbers related to each stoichiometric rate represent an average of three independent transformations. (C) Given a fixed insert to vector stoichiometric rate of 2:1, increasing amounts of the pUC19 vector was co-transformed with a proportional quantity of the fragment 3B. The average colony numbers found after each transformation assay (performed in triplicates) were plotted (red). During each cloning assay, competent cells were transformed with the pUC19 vector alone to assess the background of empty vectors (blue).

The pUC19 vector was linearized by PCR amplification. In most cases forward and reverse primers were designed to anneal them apart from each other on the pUC19 sequence (GenBank accession number M77789.2). This resulted in amplification of a simplified pUC19 backbone in which the *lac*Z gene and the multiple cloning sites were deleted (see Fig. 1). PCR amplification of the receiver plasmids was carried out from 5.0 to 10.0 ng (normal protocol) or 0.5 ng (optimized protocol) of template DNA in a 50 μL reaction volume. Insertion fragments derived from the yeast *Schizosaccharomyces japonicus* var. *versatilis* were amplified by PCR from about 30–60 ng of genomic DNA per 50 μL reaction volume. Yeast specific primers were designed based on the *S*. *japonicus* var. *japonicus* genome (GenBank accession number AATM00000000.2). The PCR fragments corresponding to the antibiotic resistance cassettes kanMX, zeoMX, natMX, and hphMX were derived from the plasmids pUG6, pUG66, pAG25, and pAG34, respectively (EUROSCARF collection) [18]. The amount of these templates used per reaction volume of 50 μL was 5–10 ng. The sequences of all oligonucleotides used in this study are listed in the supplementary S1 Table. PCRs for amplification of cloning vectors and inserts were carried out using Phusion High-Fidelity DNA Polymerase (New England Biolabs, Ipswich, MA, USA) following the manufacturer’s instructions. Optimal PCR primer annealing temperatures were chosen according to the oligonucleotide’s specific Tm or were adjusted empirically. We invariably used 30 cycles of amplification. To generate enough material for gel purification, we normally prepared PCR reactions in duplicates or triplicates. When specified in the text, after PCR we depleted the template plasmid by digesting it with 10 U of DpnI (New England Biolabs, Ipswich, MA, USA) for 1 h in the same buffer used for the PCR. Restriction endonucleases PvuII, EcoRI and HindIII were purchased from New England Biolabs (Ipswich, MA, USA) and used according to manufacturer’s instructions.

### Gel purification of PCR products

PCR products were fractionated by electrophoresis on agarose gels. In order to avoid damage to the DNA by the transilluminator UV light, we used a special procedure for gel-purification of the cloning fragments. We ran a small aliquot of the PCR product (reference DNA) in an adjacent lane to the one where most of the PCR fragment DNA to be purified was migrating in parallel. After electrophoresis, the gel was longitudinally sliced in between the parallel lanes and only the part of the gel containing the reference DNA was placed onto the transilluminator. By verifying the position of the reference DNA on the gel, we could deduce the exact location where the bulk DNA of the PCR product co-migrated. This allowed us to excise the DNA band of interest without exposing it to the UV damaging effect associated with the common procedure of cutting the gel directly on the transilluminator [19]. DNA was recovered from the agarose gel by the use of the QIAquick gel extraction kit (QIAGEN, Hilden, Germany). The amount of purified DNA was quantified using nanodrop (Life Technologies, Waltham, MA, USA) or Qubit (Life Technologies, Waltham, MA, USA). Both delivered similar quantifications when compared.

### Transformation of *E*. *coli* cells and screening of positive clones by colony PCR

All cloning procedures were carried out using the *E*. *coli* strain DH5α [20]. Competent cells were prepared according to the rubidium chloride protocol [21]. Transformation was performed by the heat shock method [21]. Briefly, we first mixed both the PCR fragments and linear vector in a 10 μL volume of pure water. This amount was transferred into a microcentrifuge tube containing 50 μL of thawed competent cells. The mixture was incubated for 20 min on ice. Heat shock was carried out by placing the tubes in a water bath for 1 min at 42°C. Readily after the heat treatment, cells were placed on ice for 5 min. For outgrowth, 240 μL of the SOC medium was added on the thermal-shocked cells, which were left to recover at 37°C for 1 h. After that time, 300 μL of cells were plated on solid LB medium containing ampicillin as selective antibiotic. Cells grew overnight at 37°C. For experiments evaluating the effect of reactant stoichiometry, amount, and overlapping length, each cloning assay was performed in triplicate transformations. Colony numbers were counted on the following day and average values were calculated. To test for positive clones, cellular material from 20 randomly chosen colonies was transferred into PCR tubes containing 50 μL of H_2_O. The tubes were boiled at 96°C for 10 min to burst the cells and release the plasmidial DNA into solution. Usually 1–3 μL of this solution was taken to amplify the inserts during 35 PCR cycles with Taq DNA polymerase (Sigma-Aldrich, St. Louis, MO, US). Positive clones were verified by agarose gel electrophoresis. Plasmidial DNA was purified from selected positive clones (QIAprep Spin Miniprep Kit, QIAGEN, Hilden, Germany) to confirm using Sanger DNA sequencing (Applied Biosystems 3130xl Genetic Analyzer, Life Technologies, Waltham, MA, USA) the correct insertion of the fragment into the vector.

## Results

### Cloning PCR fragments by homologous recombination in *E*. *coli*: proof of principle

We sought to test recombinational cloning in *E*. *coli* while assembling different antibiotic resistance cassettes to introduce into the chromosome of the yeast *Schizosaccharomyces japonicus var*. *versatiles* studied in our lab. We first aimed at amplifying and cloning a specific intergenic region on the Chromosome I (IMACS marker 3B) that has previously been demonstrated to be neutral for insertion of a kanMX cassette [22]. By targeting this genomic *locus*, we amplified a PCR product (fragment 3B, Fig. 1A) with primers containing 5′ tails overlapping the vector pUC19 at specific positions. In parallel, we linearized the pUC19 plasmid using PCR with oligonucleotides designed to have 5′ appendages homologous to the fragment 3B. As a result, both the fragment 3B (494 bp) and pUC19 (2314 bp) shared 20 nucleotides in common on their termini (Fig. 1A). After careful gel purification and with no further treatments, we transformed a mixture of both fragments in *E*. *coli*. In parallel, to check for a vector re-circularization background, we transformed competent cells alone with the linear pUC19 vector. Growth of transformed cells on solid LB medium with ampicillin resulted in hundreds of colonies only in plates corresponding to the co-transformation of PCR fragments 3B and pUC19 (data not shown). A greatly reduced number of colonies emerged on the control plates. About ten randomly picked colonies were tested by PCR, revealing 100% cloning efficiency. The correct insertion of the fragment 3B into the pUC19 vector was confirmed by Sanger sequencing. The promising results obtained during this initial trial and the simplicity of the gap-repair cloning protocol in *E*. *coli* motivated us to conduct experiments to identify parameters that might affect the efficiency of this technique.

### Stoichiometry between insert and vector

We asked whether varying the stoichiometric rate of insert to a fixed amount of vector would impact cloning yields, as measured by the number of colonies obtained after plasmid and insert co-transformation, and compared this to the transformation of the vector alone. By re-doing the previous cloning assay (Fig. 1A), we tested three variable stoichiometric amounts of the fragment 3B to a fixed 100 ng of the pUC19 (1:1, 2:1, and 4:1). In all cases, the numbers of colonies obtained after vector and insert co-transformation were significantly higher (p < 0.05, ANOVA followed by Bonferroni post-hoc test) than the vector alone (Fig. 1B). This indicates that productive formation of plasmids containing the insert is above the background of empty pUC19 vectors. However, no significant variations in colony numbers were observed between the different stoichiometric rates tested. Slightly more colonies were noted in the treatment in which the rate of insert to the vector was 2:1. Although not significant, we chose to use this ratio as a standard procedure for our cloning protocol.

### Optimal amount of insert and vector at a stoichiometric rate of 2:1

Deciding on a fixed stoichiometric rate of 2:1 between the insert and the vector, we sought to test whether different concentrations of reactants would affect cloning yields. We transformed competent cells with step-wise quantities of the pUC19 linearized vector (25, 50, 75, 100, 150, 250, and 500 ng) mixed with a corresponding amount of the PCR fragment 3B (about 11, 21, 32, 42, 64, 107, 213 ng, respectively). To assess the number of false positives, during each treatment we also transformed *E*. *coli* with the corresponding amount of the pUC19 alone. Plotting the number of colonies obtained after each transformation resulted in a graph displaying a linear correlation between colony numbers and the amount of reactants (Fig. 1C). This direct correlation was observed until 150 ng vector and 64 ng of insert were co-transformed. With higher concentrations of recombination reactants, no further increments in colony numbers were verified. Instead, an undesirable increase in vector self-circularization background was noted with higher concentrations of the linear pUC19. Optimal transformation yields were obtained in the range of 100 to 250 ng of vector. To look for positive insertion events, we tested by PCR 20 randomly picked colonies taken from the treatment conditions in which 100 ng and 150 ng of vector were co-transformed with respective amounts of inserts. In both cases, we observed 100% cloning efficiency (S1A and S1B Figs.).

### Length of overlap between the insert and vector

**Fig 2:**
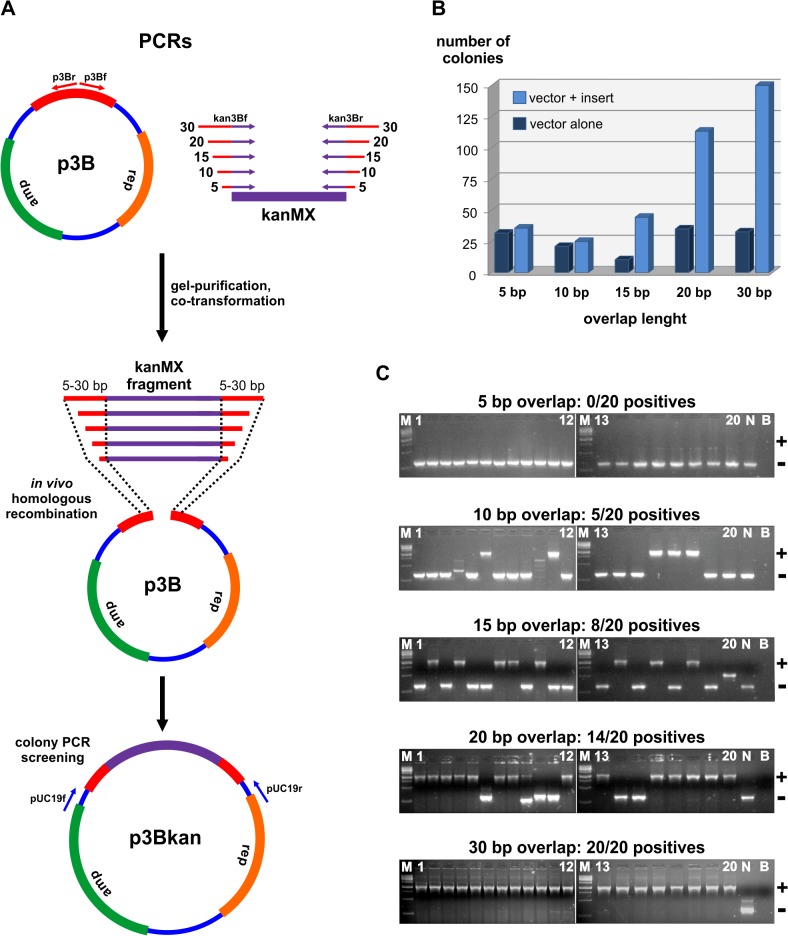
The length of overlap between the insert and vector affects cloning yields. (A) The p3B plasmid was linearized by PCR with primers p3Bf and p3Br. In parallel, the kanMX cassette was amplified by PCR using combinations of the primers kan3Bf and kan3Br containing 5′ overhangs (red tails) varying from 5 to 20 nucleotides identical to the p3B vector. PCR-amplified vector and fragments were gel-purified. Each one of the five different kanMX fragments was independently transformed in *E*. *coli* together with the p3B linear plasmid. *In vivo* homologous recombination between the p3B vector and the kanMX fragments generated the p3Bkan plasmid. (B) The number of colonies obtained increases with the length of overlap between the fragment kanMX and the linear plasmid p3B. Colony numbers represent an average value from three independent transformations. The linear p3B plasmid was transformed alone to assess the background of empty vectors during each transformation. (C) Agarose gels displaying the results of colony PCRs to check for positive insertion events in each one of the five different transformation assays. For each condition, 20 colonies were randomly chosen for PCR amplification with the primers pUC19f and pUC19r. Positive colonies (+) have a molecular weight of 2072 bp. Negative colonies (-) have the molecular weight of 643 bp. Abbreviations correspond to a molecular weight marker (M), a negative control (N) obtained by the PCR amplification of a colony taken from the transformation of the vector alone, and a PCR blank (B) control. Plasmid DNA was extracted from one positive colony and sequenced to confirm the correct insertion event.

We further tested how the length of identical flanking sequences shared between the insert and vector can influence the rate of successful recombination events. We separately assayed the cloning of five PCR fragments containing 5, 10, 15, 20, and 30 bp respectively of sequence overlap to both ends of a linearized destination plasmid (Fig. 2A). The receiver vector was prepared from the plasmid p3B (which resulted from the previous cloning experiment, Fig. 1A) by PCR amplification with the primers p3Bfor and p3Brev (Fig. 2A). The five different insert fragments were obtained by PCR amplification of the kanMX cassette with oligonucleotides containing 5′ tails of variable lengths of identity (from 5 to 30 nucleotides) to the p3B vector. The co-transformation of the kanMX fragments and p3B linear plasmid resulted in the construct p3Bkan (Fig. 2A), harboring an insertion cassette designed to be integrated into the chromosome I of *S*. *japonicus*. The average numbers of colonies obtained for each of the five independent transformation assays showed that a direct correlation exists between recombination frequency and the length of insert to vector overlap (Fig. 2B). Accordingly, a 30 bp sequence match between vector and insert termini yielded the highest number of colonies. Using colony PCR, we screened the rate of positive insertion events in 20 randomly picked colonies from each of the five different cloning assays (Fig. 2C). The results revealed that the number of positive clones is also proportional to the extent of identity between the insert and linear plasmid. A 10 bp overlap seems to be a minimal requirement for the kanMX insert to recombine with the p3B plasmid (5 positives out of 20), while terminal sequence tracts of 30 bp identical to the vector resulted in 100% positive insertion events (Fig. 2C, gel at the bottom). Inserts with 20 bp long homology tracts to the vector rendered 70% of positive clones. For routine cloning, an insert to vector identity length of 20 bp might be favorable for practical and economic reasons. In this case, cloning yields higher than 70% are possible if further improvements are added to increase the efficiency of the protocol (below).

### The optimal cloning of five different PCR fragments

**Fig 3:**
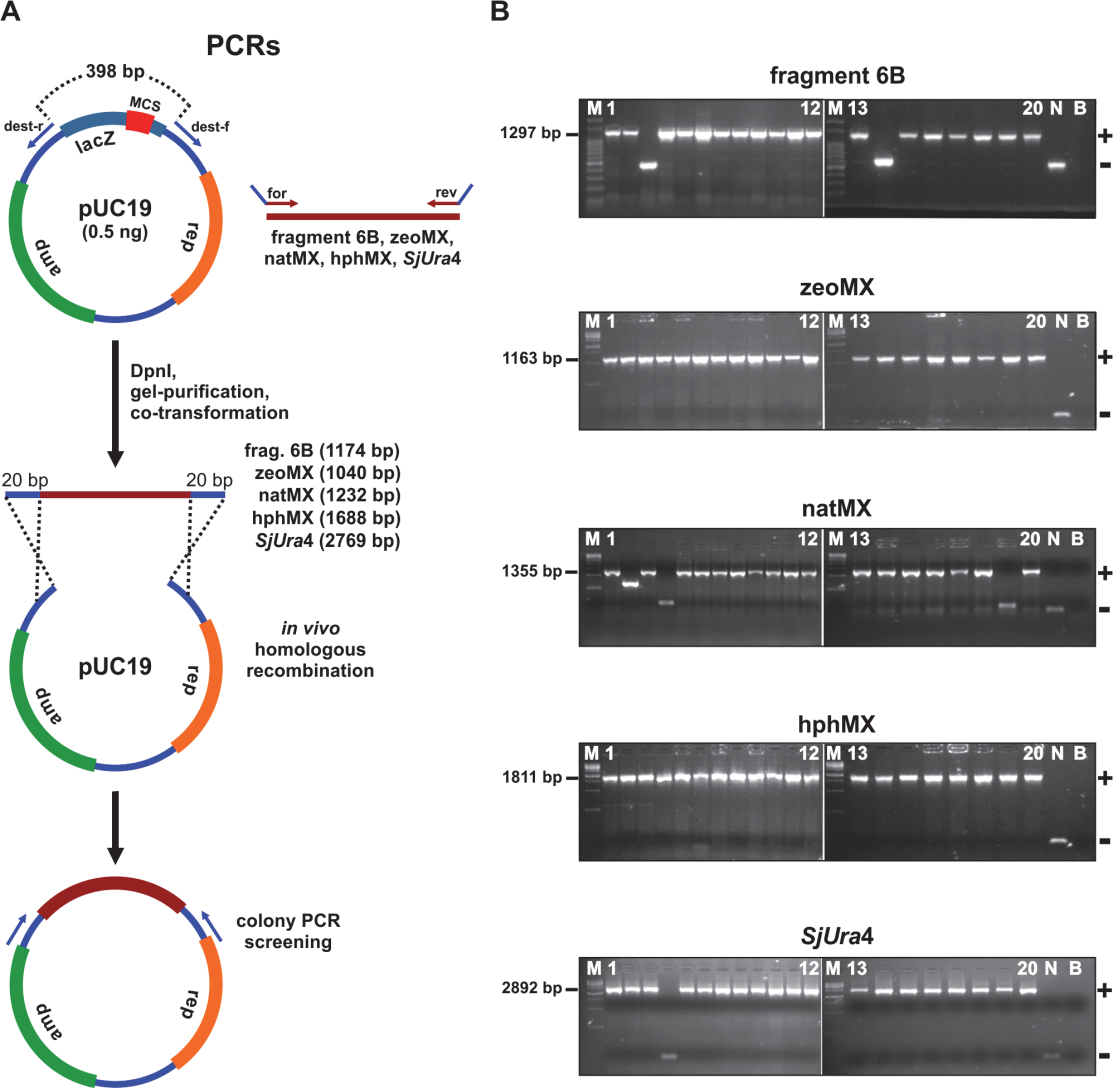
The test of an optimized recombinational cloning protocol. (A) 0.5 ng of the pUC19 vector was amplified by PCR with the primers dest-f and dest-r. A sequence gap of 398 bp separates the 5′ termini of those primers. Five different templates were amplified with specific primers containing 20 bp long 5′ tails identical to the respective ends of the linear pUC19 vector. The *S*. *japonicus* region 6B was amplified by PCR with primers 6Bf and 6Br, while the cassettes zeoMX, natMX, and hphMX were produced with the primers MXf and MXr. Finally, the *S*. *japonicus Ura*4 gene was amplified with the primers Ura4f and Ura4r. The vector and PCR fragments were treated with DpnI and co-transformed in *E*. *coli* cells. Homologous recombination of each of these fragments with the pUC19 destination vector resulted in a different recombinant plasmid. (B) After transformation of each of the five constructs, 20 colonies were randomly picked to check for positive cloning events (+) by PCR with the primers pUC19f and pUC19r. The sequencing of one plasmid corresponding to each different construct confirmed a positive cloning event. Abbreviations are as described in Fig. 2.

So far we have empirically established important parameters that affect recombinational cloning efficiency (Table 1). These are the use of 100 to 250 ng of vector and the corresponding amount of fragment adjusted to fit an insert to vector ratio of 2:1 (Fig. 1B and 1C). In addition, higher cloning yields are achieved when inserts have 20 or more nucleotides overlapping the vector (Fig. 2B and 2C). There are further optimization parameters that can improve cloning efficiently by decreasing the undesirable background of empty vectors (Table 1). In cloning protocols that use PCR-amplified vectors, false positive colonies are often a consequence of the template plasmids being carried over from the PCR reaction into the transformation [15,16]. An advantageous fact is that in most cases the plasmid used as a template was previously methylated *in vivo* during its propagation in *E*. *coli* before DNA extraction. Therefore, after the PCR, the plasmid template can be selectively destroyed by using the methylation sensitive DpnI endonuclease, which will leave intact the non-methylated PCR-amplified vector [15,16]. The use of DpnI is also recommended when a plasmid is the template to produce insertion fragments by PCR. Another critical parameter that might be important to observe when cloning vectors are prepared by PCR is the disposal of primers over the plasmid template. It has been suggested that if the annealing positions of the two PCR primers are relatively close together on the template plasmid, primer extension by DNA polymerase can progress all the way around the plasmid. This linear extension might result in nicked copies of the template plasmid that increases the background of empty vectors after transformation (discussed in ref. [15]). We noticed that when we amplified the vector using PCR with primers that were distally placed (see Fig. 1A), we obtained 100% efficiency in cloning PCR products with 20 bp flanks of homology to the vector (S1A and S1B Figs.). However, when primers were juxtaposed on the template sequence (Fig. 2A), the cloning efficiency of fragments with the same length of homology to the vector dropped to 70% (Fig. 3B, second gel from the bottom). Accordingly, Stevenson and coworkers demonstrated that an increased frequency of positive colonies is achieved when a larger gap in between the 5′ ends of the primers is introduced [15]. In addition, they showed that an effective way to reduce nicked vector background and template plasmid carryover during transformation is to use a minimal amount of 0.5 ng template vector per reaction (Table 1).

**Table 1:** Critical parameters affecting gap-repair cloning efficiency in *E*. *coli*.

Parameters	Comments
Fragment:vector stoichiometry of 2:1.	Not really significant (Fig. 1B). Other reports suggest stoichiometric rates of 2.5:1 [15] and 1:1 [16].
The use of 100–250 ng of vector and the corresponding amount of fragment to fit an insert:vector stoichiometry of 2:1.	Best colony forming numbers are obtained with 150 ng of vector; however, the background of empty vectors also increases (Fig. 1C). 100 ng of the vector gives excellent results with a reduced negative background (Figs. 1C and 3).
20–30 bp of overlap between the insert and vector.	Cloning yields are positively correlated to the length of overlap. A 20 bp match between the vector and insert gives excellent rates of positive insertion events (S1A and B Fig; Fig. 3B).
The use of DpnI after PCR amplification of the vector.	Digestion of the template plasmid by DpnI precludes its carryover to the transformation step of the protocol, thereby minimizing the background of empty vectors [15,16].
The disposition of a large gap in between the 5′ termini of primers when amplifying the vector by PCR.	This minimizes the linear amplification of the vector which might result in nicked copies of the template vector (nicked vector background) [15].
The use of 0.5 ng of template plasmid when linearizing the vector by PCR amplification.	This serves to minimize the carryover of both the template and nicked plasmids from the PCR to the transformation step of the protocol [15].
Linearization of the vector by digestion with restriction endonucleases.	Useful when the vector is too large to be amplified by PCR. It this case the digestion with two restriction endonucleases and the dephosphorylation of the vector might minimize vector re-circularization [10,11,13].
The co-transformation of vector and inserts directly from the PCR reaction, without gel purification.	It’s a time-sparing procedure [15,16]. It’s possible whenever the PCR product displays a clear single band on the agarose gel electrophoresis.

We sought to test an optimized gap-repair protocol by combining the above discussed improvements during the cloning of five different PCR fragments (Fig. 3). In this optimized protocol, the receiver plasmid was amplified by PCR with the use of forward and reverse primers disposed 398 bps apart on the pUC19 sequence (Fig. 3A). This generated a 2278 bps linear PCR fragment of the vector. The amount of pUC19 template used was 0.5 ng per 50 uL PCR reaction. To digest the plasmid template after PCR amplification, we incubated the PCR solution with 10 units of DpnI for 1 h. Several reactions were combined and gel purified, generating enough material for multiple cloning assays. All inserts were prepared by PCR with primers containing 5′ appendages of 20 nucleotides overlapping the respective ends of the linear pUC19 plasmid (Fig. 3A). By following an insert to vector stoichiometric rate of 2:1, the amount of the different inserts was adjusted to fit 100 ng of vector. The first PCR fragment to be cloned was amplified from the *S*. *japonicus* genome with primers specific to the region of the marker IMACS 6B (fragment 6B of 1174 bp) [22]. Following the vector and insert co-transformation, we detected using colony PCR that fragment 6B was successfully inserted into the vector in 18 out of 20 colonies tested (Fig. 3B, first gel). The next three insertion fragments generated by PCR (Fig. 3A) corresponded to the antibiotic insertion cassettes zeoMX (1040 bp), natMX (1232 bp), and hphMX (1688 bp). The cassettes were PCR amplified from template plasmids that were DpnI digested after the PCR reaction. Co-transformation of fragments zeoMX and hphMX with the destination vector resulted in 100% cloning efficiency (Fig. 3B, second and forth gels), whereas homologous recombination of the natMX cassette with the pUC19 vector was successful in 17 of the 20 colonies tested (third gel on Fig. 3B). A final insert fragment of 2769 bp was generated by PCR amplification of the whole *Ura*4 gene (including the CDS, promoter, terminator, and flaking sequences) from the genome of *S*. *japonicus* (Fig. 3A). Cloning of the *SjUra*4 fragment resulted in 19/20 positives colonies (Fig. 3B, gel at the bottom). From a total 100 colonies checked by PCR, 94 corresponded to positive insertion events. Therefore, our optimized protocol delivered an excellent rate of 94% cloning efficiency.

### Simultaneous cloning of two PCR fragments into a vector

**Fig 4:**
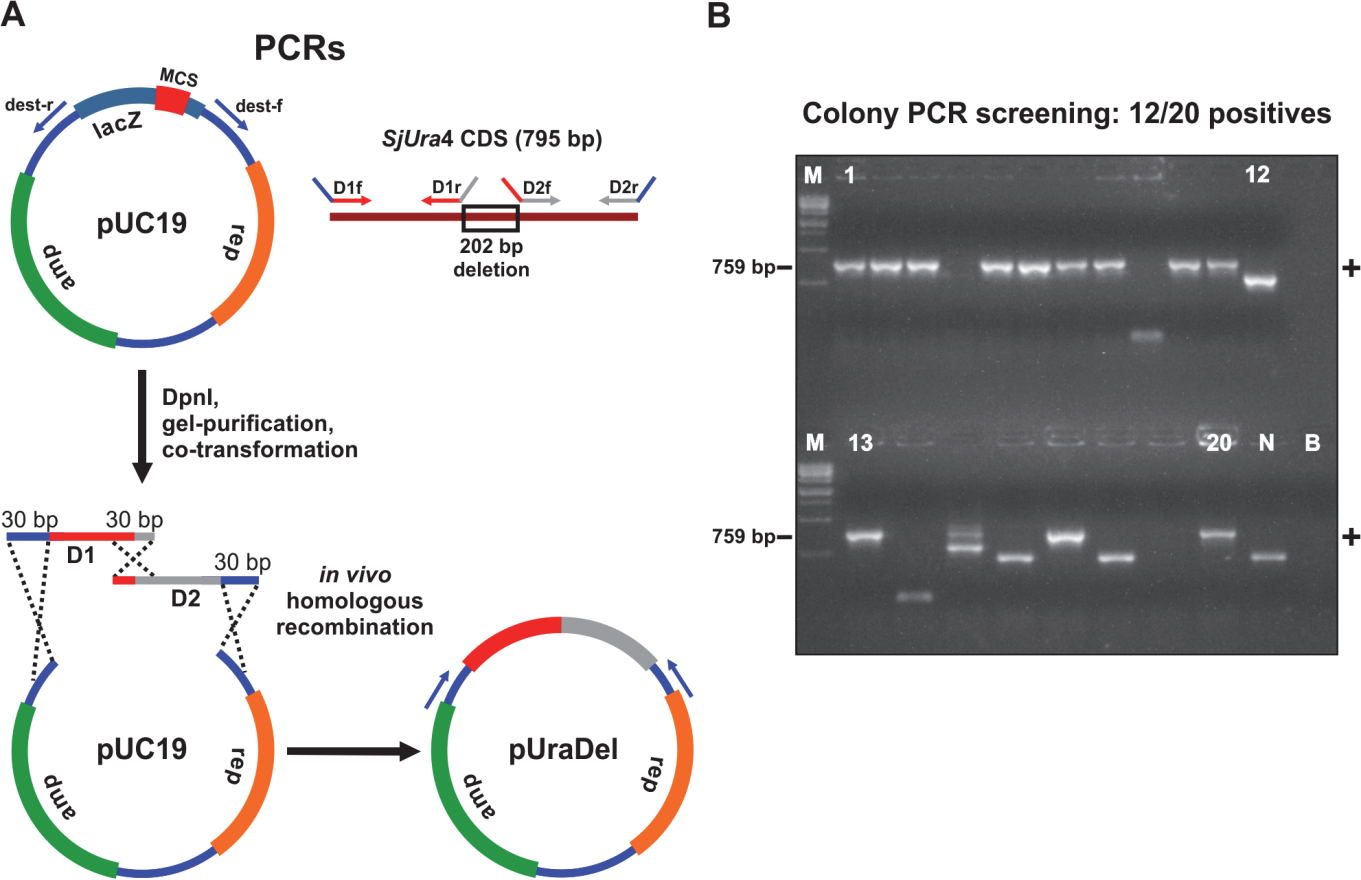
The simultaneous cloning of two PCR products into the pUC19 plasmid. (A) The vector pUC19 was prepared by PCR with the primers dest-f and dest-r. The fragment D1 (red) was generated by PCR amplification of the *SjUra*4 5′ region with the primers D1f and D1r. The fragment D2 (gray) was generated by PCR amplification of the *SjUra*4 3′ region with the primers D2f and D2r. Primers were designed to generate a 30 bp overlap between the D1 and D2 fragments, and a 30 bp match of these inserts with the respective termini of the pUC19 vector. Homologous recombination of the D1, D2, and pUC19 fragments generated the plasmid pUraDel, which corresponded to *SjUra*4 CDS containing a 202 bp internal deletion. (B) PCR screening of 20 randomly chosen colonies with the primers pUC19f and pUC19r. PCR products corresponding to 12 positive colonies (+) had the expected molecular weight of 759 bp. The correct size was confirmed by sequencing a recombinant plasmid extracted from one positive colony. Abbreviations are as described in Fig. 2.

The results obtained so far clearly demonstrate that homologous recombination is extremely efficient to clone single PCR fragments. We further asked whether recombinational cloning would be a practical method for assembling into a vector two PCR fragments at once. We especially aimed to construct a deletion cassette of the *S*. *japonicus Ura*4 gene that would suit our purpose of generating uracil auxotrophic yeast mutants [23]. To produce a 202 bp deletion in the middle of the *SjUra*4 CDS, we amplified two PCR products corresponding to the 5′- (D1 fragment of 334 bp) and the 3′-part (D2 product of 344 bp) of this gene (Fig. 4A). Those two PCR fragments were intended to be directionally cloned as a fusion into our previously prepared pUC19 destination vector. In order to stimulate homologous recombination between D1 and D2, we designed primers to generate PCR products that have 30 bp overlap (Fig. 4A). In addition, the D1 fragment carried a sequence tail of 30 bp identical to one end of the pUC19 vector, whereas D2 had a 3′ extension of 30 nucleotides matching the other extremity of the pUC19 vector (Fig. 4A). The two *SjUra*4 fragments were co-transformed with 100 ng of the pUC19 vector following a 2:2:1 stoichiometry. The resulting colonies were screened by PCR to find positive insertion events (Fig. 4B). From a total of 20 inspected colonies, 12 had an insert of the expected size, confirming that in 60% of the cases a positive cloning occurred. This result demonstrated that recombinational cloning in *E*. *coli* is an excellent option to generate a fusion of two fragments that can be useful to produce deletions, insertions, or site-directed mutagenesis of a targeted gene.

### Gap-repair cloning in *E*. *coli* does not require ssDNA ends on the cloning reactants

The PIPE [14] and FastCloning [16] protocols propose simple cloning procedures that in essence follow the same principle of the recombinational method originally described in 1993 [10,11] and elaborated in this work. That is, when PCR fragments and vectors containing flanking sequence tracts of homology are co-transformed in *E*. *coli*, they are naturally assembled *in vivo* as a recombinant plasmid. However, the authors of the PIPE protocol put forward an alternative explanation for the cloning mechanism that contrasts with the homologous recombination phenomenon favored here. Their interpretation is based on the assumption that the DNA polymerase produces incomplete extensions of the primers, leaving 5′ ssDNA termini in a substantial fraction of PCR products [14]. As both vector and insert are amplified by PCR during the PIPE protocol, the complementary ssDNA overhangs anneal, forming a hybrid plasmid with nicks, which is repaired *in vivo* after transformation [14]. A similar explanation is also favored by the authors of the FastCloning protocol who suggest that fortuitous 3′ to 5′ exonuclease activity of the DNA polymerase leaves complementary ssDNA ends when vector and inserts template are digested by DpnI [16].

**Fig 5:**
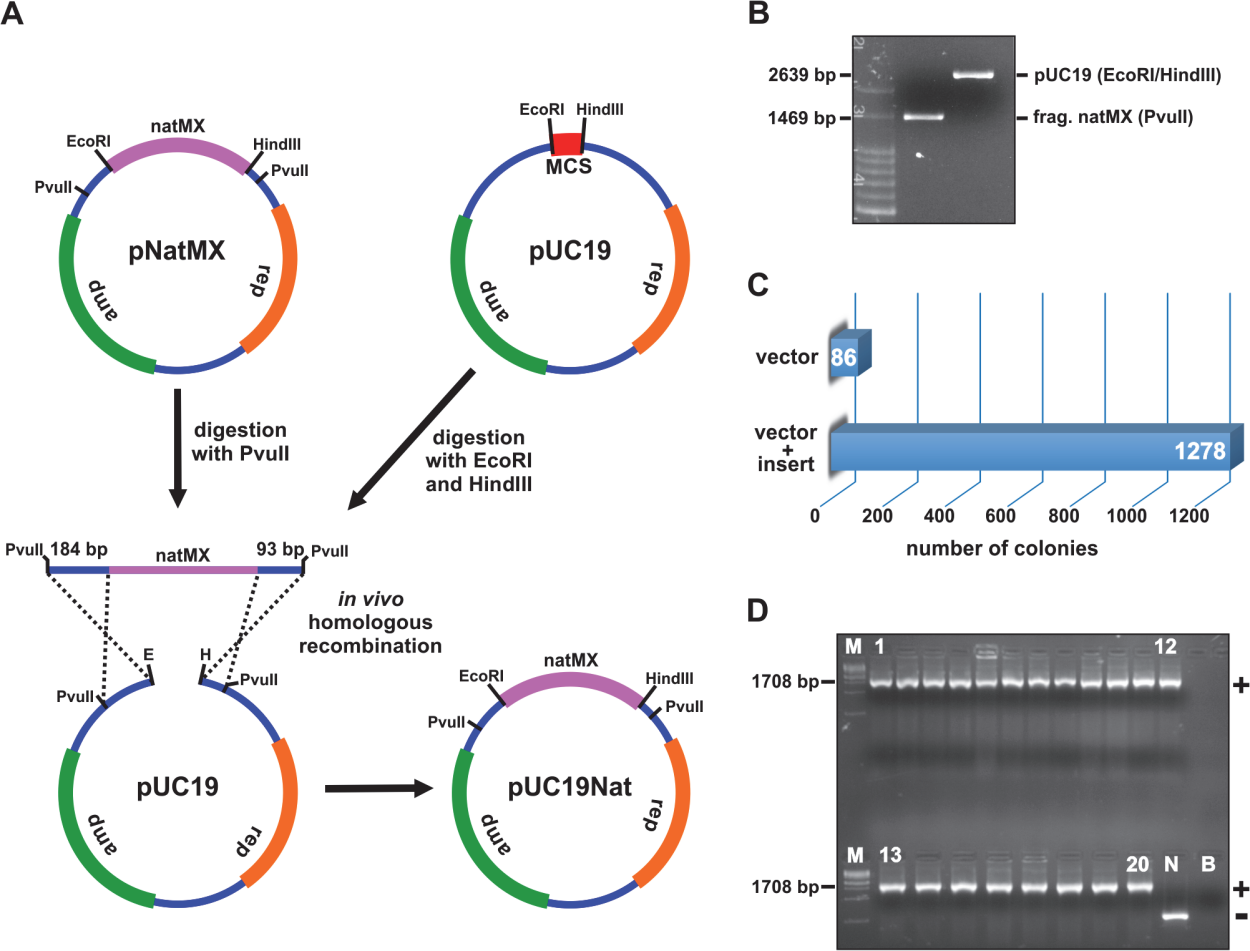
Homologous recombination between vector and insert generated by restriction endonucleases. (A) The pNatMX was cleaved with the PvuII endonuclease generating the natMX fragment of 1469 bp. The pUC19 was prepared by digestion with the EcoRI and HindIII restriction enzymes, resulting in a 2639 bp linear plasmid. Homologous recombination between the natMX and pUC19 fragments generated the pUC19Nat plasmid. (B) Agarose gel electrophoresis after gel purification of the fragments natMX and pUC19. (C) The counting of colonies after transformation of the vector alone and co-transformation of the pUC19 plus the fragment natMX. (D) Colony PCR screening confirmed 100% positive cloning events. Abbreviations are as described in Fig. 2.

However, these interpretations are readily challenged by feasible gap-repair cloning of PCR products into plasmids linearized with restriction enzymes (refs. [10,11,13] and also our personal observations). To obtain decisive insight into the question of whether PCR-generated ssDNA termini on both vector and inserts are really critical to gap-repair cloning, we devised an experiment in which the cloning reactants are fully prepared with restriction enzymes. In preliminary cloning, we inserted a PCR product corresponding to the natMX cassette into the PCR-amplified pUC19 vector (S2 Fig.). This resulted in the pNatMX plasmid. With the help of the PvuII endonuclease, we then cut a 1469 bp blunt-ended DNA fragment from the pNatMX plasmid (Fig. 5A and second lane on the gel at Fig. 5B). This contained the natMX cassette flanked by 184 and 93 bp long sequences identical to the pUC19 vector (Fig. 5A). In parallel, we digested the pUC19 vector with the EcoRI and HindIII endonucleases (Fig. 5A and third lane on the gel of Fig. 5B). It should be noted that exempting short overhangs of four nucleotides left on the vector by the EcoRI and HindIII endonucleases, both vector and insert constitute dsDNA molecules. After gel purification of the restriction fragments (Fig. 5B), we co-transformed the PvuII natMX insert and the EcoRI/HindIII vector into *E*. *coli*. Concomitantly, we transformed competent cells with the linear pUC19 vector to assess the background of plasmid re-circularization. As a result, the next day we counted 1278 colonies on plates corresponding to the vector and fragment co-transformation (Fig. 5C). In sharp contrast, only 86 colonies appeared in plates relative to the transformation of the vector alone. Using colony PCR, we confirmed that 100% of colonies tested contained the insert (Fig. 5D). This result decisively establishes that gap-repair cloning can occur when vector and inserts correspond to dsDNA molecules.

## Discussion

Cloning by homologous recombination in *E*. *coli* was independently reported by two groups in the year 1993 [10,11]. Since then this method has not reached wide popularity among molecular biologists. This is surprising, considering that recombinational cloning offers substantial advantages in terms of time saving, lower costs, and increases in efficiency when compared to traditional ligase-dependent protocols. Therefore, the dissemination of the gap-repair method in *E*. *coli* might benefit an ample community of molecular biologists still tied up to conventional cloning practices. One important step to spread the use of recombinational cloning in *E*. *coli* is to resolve contradicting interpretations on how the technique essentially works. This is pertinent in light of the assumptions that DNA polymerase generated ssDNA overhangs on both the vector and insert might be critical for gap-repair to occur [14,16]. Here we show that vector and inserts prepared with restriction endonucleases can easily recombine *in vivo* after transformation (Fig. 5), which is in agreement with previous reports demonstrating the insertion of PCR fragments into plasmids linearized with restriction enzymes [10,11,13]. Although these results rule out the critical need of ssDNA ends on the reactants prior to the transformation, it is still possible that following the heat shock dsDNA ends can be processed by the cellular machinery into ssDNA, thereby facilitating the *in vivo* annealing of vector and insert. Despite this speculation, the most likely interpretation is that a homologous recombination reaction is at the heart of the cloning process involving co-transformation and *in vivo* assembly of molecules containing overlapping ends. That is the same mechanistic principle operating in the analogous gap-repair approach in yeast [8,9]. However, the DH5α strain used in our study is *rec*A negative [20], suggesting that a non-conventional homologous recombination mechanism might be involved. In fact, a *rec*A-independent homologous recombination pathway, which is characterized by operating in substrates having limited stretches of homology, has been uncovered in *E*. *coli* [24,25]. In specific recombination assays, this *rec*A-independent route is the only one responsible for recombining plasmids with 25 bp homology [24], a similar stretch of identity used in our cloning experiments. Interestingly, this homologous recombination pathway is enhanced in the absence of the *rec*A protein and cellular exonucleases [25]. An analogous increase in recombination efficiencies for cloning PCR fragments having 42 bp overlap with the vector ends was observed during the SLiCE procedure when reactants were incubated on cell extracts of *rec*A negative strains [2]. It would be of interest to elucidate whether this cryptic homologous recombination pathway in *E*. *coli* (or another one) might be responsible for the homology-based cloning of PCR fragments described in this study.

Another important factor to popularize the gap-repair method in *E*. *coli* is the optimization of the cloning procedure. This work and others provided a set of parameters (summarized in Table 1) that might be useful as guidance to researchers aimed at designing their own protocol. We tested five of these parameters in a series of cloning assays involving the insertion of different PCR products into a common destination vector also prepared by PCR (Fig. 3). Those are: (i) The PCR amplification of inserts having 20 bp long sequence tracts identical to the vector ends; (ii) The design of primers whose 5′ ends are distantly positioned on the pUC19 vector [15]; (iii) The PCR amplification of the vector with an amount of 0.5 ng plasmid template per 50 uL reaction [15]; (iv) The DpnI digestion of the plasmid template used for vector PCR amplification [15,16]; (v) The use of 100 ng vector in combination with the necessary amount of PCR product to fit a molar ratio of 2:1. During this cloning series, we obtained 94 positive colonies out of 100 tested (Fig. 3B), which is already an excellent benchmark for any cloning procedure. However, we anticipate that further improvements in this protocol are yet still possible. We observed that the cloning efficiency increased proportionally to the length of overlap between the insert and vector (Figs. 2B and 2C). In our standard protocol, we choose to use 20 bp long homologous regions, which is an option for combining good cloning efficiency with affordable primer synthesis costs. However, the amplification of inserts with flanking tracts of homology longer than 20 bp (e.g., 30 bp or more) are expected to increase the number of successful recombination events. Another possibility is to add more space in between the primers used to amplify the vector. This can even further minimize the background of empty vectors linearly amplified during PCR [15]. To prepare the pUC19 vector by PCR, we used 398 bp distal primers (Fig. 3A), which is already close to the limit we could set for that plasmid. A better option might be the PCR amplification of the destination vector starting from a plasmid already containing a large insert in between the pair of primers. One excellent possibility is the use of a counter-selection marker (e.g., the *ccdB* gene) as an interspersing DNA in between the primers [14]. This will effectively eliminate any transformed *E*. *coli* cell that has received a copy of the template plasmid.

Gap-repair cloning in *E*. *coli* is not only a superior method when compared to conventional DNA recombinant techniques; it also presents a key advantage over other ligase-independent cloning approaches. As opposed to the gateway [3], SLIC [4], USER [5], OEC [6], and CPEC [7] methods, recombinational cloning in *E*. *coli* requires no further enzymatic step after PCR amplification and purification of the vector and inserts. It is therefore the fastest ligase-independent cloning method available. In this regard it is comparable to gap-repair cloning in yeast. While results in yeast are retrieved after usually 3 days after transformation [8], *E*. *coli* recombinant colonies are obtained after overnight incubation, allowing a faster isolation of greater amounts of DNA. Gap-repair cloning in *E*. *coli* is also an excellent option for assembling two PCR fragments simultaneously into a plasmid (Fig. 4). This procedure can be useful for site-directed mutagenesis and quick generation in a single step of deletions and insertions in a gene. It is also a practical solution for assembly of any bi-partite chimeric construct. However, recombinational cloning in *E*. *coli* performs poorly when it fuses more than two fragments (our personal observations). In this case, homologous recombination in yeast [26] and SLIC [4], USER [5], and CPEC [7] methods in *E*. *coli*, are preferable. Gap-repair cloning in *E*. *coli* is suited to several DNA recombinant applications. We demonstrate the assembly of a kanMX insertion cassette containing flanking sequences for integration by homologous recombination into the *S*. *japonicus* yeast chromosome (Figs. 1 and 2). Such chimeric constructs can be generated in a two-step cloning process. First, a DNA of interest is inserted into a vector (Fig. 1). Second, the resulting plasmid is opened by PCR amplification at any position to subsequently insert the desired piece of DNA (Fig. 2). This procedure can be used to produce 5′ or 3′ in-frame fusions of any kind. The same logic serves to generate deletions inside of a given gene. Such simplicity to produce PCR fragments in-frame with a reporter gene or epitope-tagged proteins encoded in a vector makes gap-repair cloning in *E*. *coli* a powerful tool for high-throughput DNA recombinant production (e.g., refs. [13] and [14]). Recombinational cloning can also be easily adapted to protocols dispensing vector and fragments gel purification, allowing implementation of a speedy cloning routine (e.g., the PIPE [14] and FastCloning [16] protocols). In this new era of synthetic biology when quick and efficient cloning methods are demanded, homologous recombination in *E*. *coli* is perhaps the fastest and most effective way for cloning up to two PCR fragments. For this reason, we believe that gap-repair cloning in *E*. *coli* has an excellent potential for being definitively integrated into the DNA recombinant tool kit of modern molecular biologists.

## Supporting Information

S1 Table PCR oligonucleotides used in this work. PCR primers are listed following the order of their citation in the text.
(DOCX)

S1 Fig Colony PCR of the p3B plasmid. Screening of 20 recombinant colonies after co-transformation of *E*. *coli* with (A) 100 ng or (B) 150 ng of the pUC19 vector, and a corresponding stoichiometric amount of the insert 3B. PCR amplification of the colonies with the primers 3Bf and 3Br gives a 494 bp band when the fragment 3B was correctly inserted (+). The sequencing of a plasmid extracted from one clone confirmed the correct insertion size. A PCR blank (B) control was loaded onto the last lane of each gel.
(TIF)

S2 Fig Cloning of the natMX cassette into the pUC19 plasmid. (A) The pUC19 vector was amplified by PCR with the primers pUCNf and pUCNr. The cassette natMX was amplified with the primers natf and natr. Homologous recombination of the two PCR fragments generated the plasmid pNatMX. (B) Colony PCR screening of 20 randomly picked colonies identified 17 positive colonies with PCR bands of the expected 1708 bp size (+). Sequencing of a plasmid corresponding to one positive colony confirmed the correct insertion of the fragment natMX into the pUC19 vector. Abbreviations are as described in Fig. 2.
(TIF)
