# Supplementary material for: Optimal Cloning of PCR Fragments by Homologous Recombination in Escherichia coli
Source: PLoS One. 2015 Mar 16;10(3):e0119221. doi: 10.1371/journal.pone.0119221 (PMC4361335; doi:10.1371/journal.pone.0119221)
Supplement: S1 Table — PCR primers are listed following the order of their citation in the text. (DOCX) [file pone.0119221.s001.docx]

| **S1 Table. PCR oligonucleotides used in this work.** | |
| --- | --- |
| pUC3Bf | CCAAACTGGATCCGCGCAGCCTGAATGGCGAATGG |
| pUC3Br | CGTGGGGGGATCCGGAGAGGCGGTTTGCGTATTGGG |
| 3Bf | GCTGCGCGGATCCAGTTTGGCAACTCTGAATGG |
| 3Br | CCTCTCCGGATCCCCCCACGACTCGTCTCTCTTTGT |
| p3Bf | AGCTACTACATTAAATGTGCC |
| p3Br | TTTTAAGATTAAGAAAGTGCATCC |
| Kan3Bf-5 | TAAAAAGCTTGCCTCGTCCCCGCC |
| Kan3Bf-10 | AATCTTAAAAAGCTTGCCTCGTCCCCGCC |
| Kan3Bf-15 | TTCTTAATCTTAAAAAGCTTGCCTCGTCCCCGCC |
| Kan3Bf-20 | GCACTTTCTTAATCTTAAAAAGCTTGCCTCGTCCCCGCC |
| Kan3Bf-30 | CGTTTGGGATGCACTTTCTTAATCTTAAAAAGCTTGCCTCGTCCCCGCC |
| Kan3Br-5 | TAGCTTCGACACTGGATGGCGGCGTT |
| Kan3Br-10 | TGTAGTAGCTTCGACACTGGATGGCGGCGTT |
| Kan3Br-15 | TTTAATGTAGTAGCTTCGACACTGGATGGCGGCGTT |
| Kan3Br-20 | GCACATTTAATGTAGTAGCTTCGACACTGGATGGCGGCGTT |
| Kan3Br-30 | TGCCGCGTTGGCACATTTAATGTAGTAGCTTCGACACTGGATGGCGGCGTT |
| pUC19f | CCGCTCGCCGCAGCCGAACGACCGAGCGC |
| pUC19r | CCATATGCGGTGTGAAATACCGCAC |
| dest-f | GCGCAGCCTGAATGGCGAATGG |
| dest-r | GGAGAGGCGGTTTGCGTATTGGG |
| 6Bf | AATACGCAAACCGCCTCTCCGTGATACTTAGGGTCCTTGG |
| 6Br | attcgccattcaggctgcgcGGTCGCGAACGCGAGCAATGG |
| MXf | AATACGCAAACCGCCTCTCCAGCTTGCCTCGTCCCCGCC |
| MXr | attcgccattcaggctgcgcTCGACACTGGATGGCGGCGTT |
| Ura4f | AATACGCAAACCGCCTCTCCCCACCGACTTTCCTATCGCC |
| Ura4r | attcgccattcaggctgcgccgtcgatgtcagctctgccc |
| D1f | AAGAGCGCCCAATACGCAAACCGCCTCTCCATGTCGGACATCGCCTTGA |
| D1r | CAATTCCAATGTGGCTCGGCAAACTTGCGATCCTC |
| D2f | TCGCAAGTTTGCCGAGCCACATTGGAATTGGCTGAG |
| D2r | atcaggcgccattcgccattcaggctgcgcTTACTGCTTGGAAATGCGA |
| pUCNf | GAATTCACTGGCCGTCGTTTTAC |
| pUCNr | AAGCTTGGCGTAATCATGGTCA |
| natf | ACCATGATTACGCCAAGCTTAGCTTGCCTCGTCCCCGCC |
| natr | AAACGACGGCCAGTGAATTCTCGACACTGGATGGCGGCGTT |
