# Supplementary figures and images for: Optimal Cloning of PCR Fragments by Homologous Recombination in Escherichia coli
Source: PLoS One. 2015 Mar 16;10(3):e0119221. doi: 10.1371/journal.pone.0119221 (PMC4361335; doi:10.1371/journal.pone.0119221)

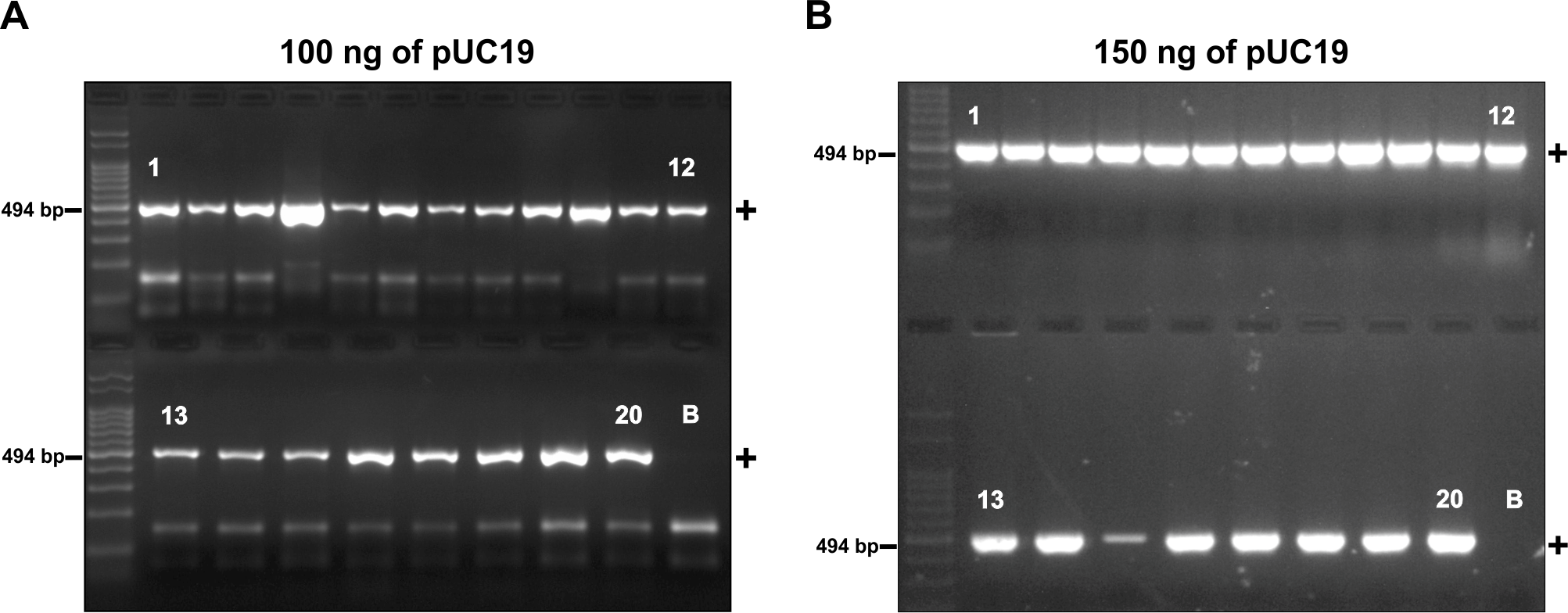

Supplement: S1 Fig — Screening of 20 recombinant colonies after co-transformation of E. coli with (A) 100 ng or (B) 150 ng of the pUC19 vector, and a corresponding stoichiometric amount of the insert 3B. PCR amplification of the colonies with the primers 3Bf and 3Br gives a 494 bp band when the fragment 3B was correctly inserted (+). The sequencing of a plasmid extracted from one clone confirmed the correct insertion size. A PCR blank (B) control was loaded onto the last lane of each gel. (TIF) [file pone.0119221.s002.tif]

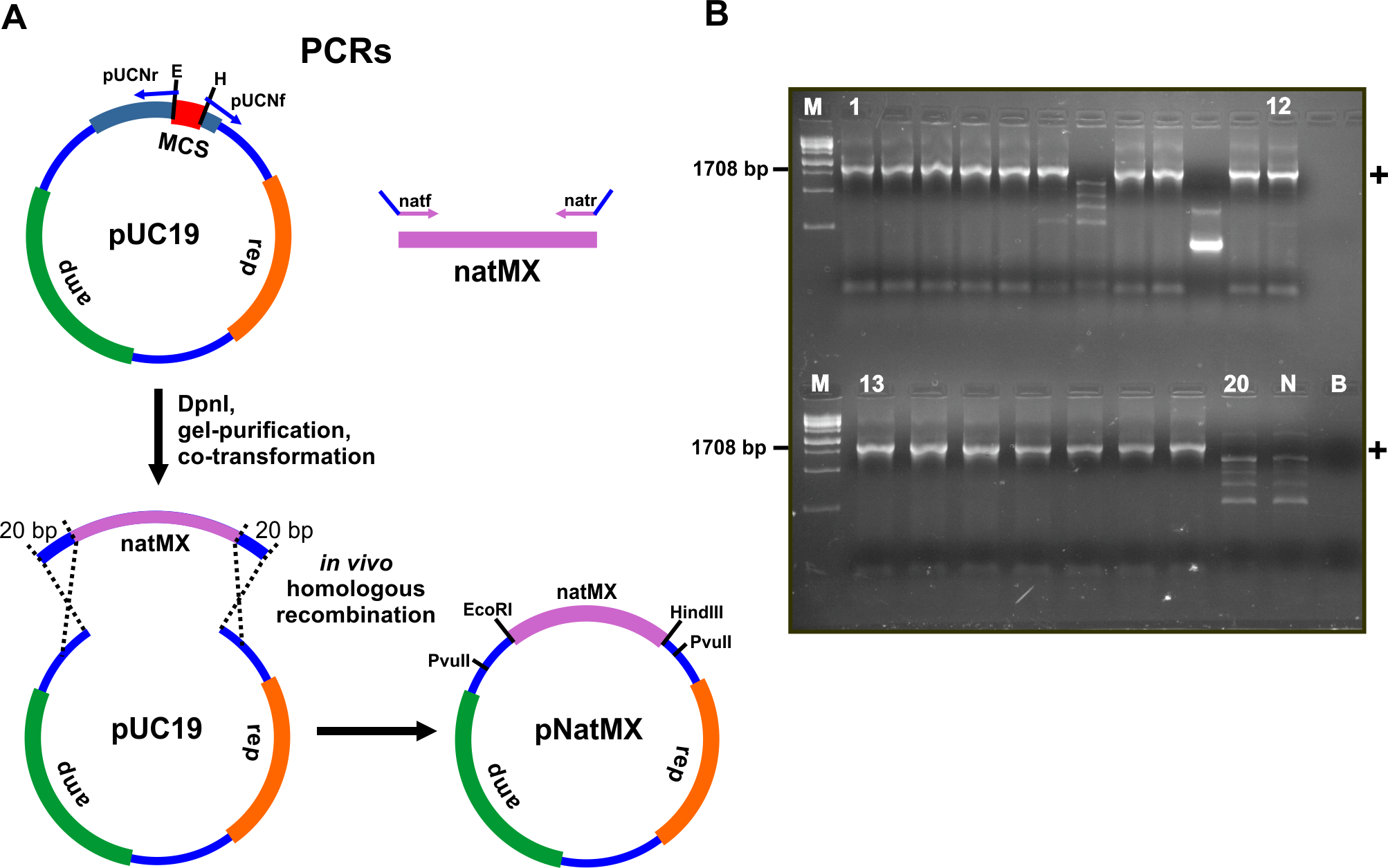

Supplement: S2 Fig — (A) The pUC19 vector was amplified by PCR with the primers pUCNf and pUCNr. The cassette natMX was amplified with the primers natf and natr. Homologous recombination of the two PCR fragments generated the plasmid pNatMX. (B) Colony PCR screening of 20 randomly picked colonies identified 17 positive colonies with PCR bands of the expected 1708 bp size (+). Sequencing of a plasmid corresponding to one positive colony confirmed the correct insertion of the fragment natMX into the pUC19 vector. Abbreviations are as described in Fig. 2. (TIF) [file pone.0119221.s003.tif]
